# Synchronization of Developmental Processes and Defense Signaling by Growth Regulating Transcription Factors

**DOI:** 10.1371/journal.pone.0098477

**Published:** 2014-05-29

**Authors:** Jinyi Liu, J. Hollis Rice, Nana Chen, Thomas J. Baum, Tarek Hewezi

**Affiliations:** 1 Department of Plant Sciences, University of Tennessee, Knoxville, Tennessee, United States of America; 2 Department of Plant Pathology and Microbiology, Iowa State University, Ames, Iowa, United States of America; Instituto de Biología Molecular y Celular de Plantas, Spain

## Abstract

Growth regulating factors (GRFs) are a conserved class of transcription factor in seed plants. GRFs are involved in various aspects of tissue differentiation and organ development. The implication of GRFs in biotic stress response has also been recently reported, suggesting a role of these transcription factors in coordinating the interaction between developmental processes and defense dynamics. However, the molecular mechanisms by which GRFs mediate the overlaps between defense signaling and developmental pathways are elusive. Here, we report large scale identification of putative target candidates of Arabidopsis GRF1 and GRF3 by comparing mRNA profiles of the *grf1/grf2/grf3* triple mutant and those of the transgenic plants overexpressing miR396-resistant version of *GRF1* or *GRF3*. We identified 1,098 and 600 genes as putative targets of GRF1 and GRF3, respectively. Functional classification of the potential target candidates revealed that GRF1 and GRF3 contribute to the regulation of various biological processes associated with defense response and disease resistance. GRF1 and GRF3 participate specifically in the regulation of defense-related transcription factors, cell-wall modifications, cytokinin biosynthesis and signaling, and secondary metabolites accumulation. GRF1 and GRF3 seem to fine-tune the crosstalk between miRNA signaling networks by regulating the expression of several miRNA target genes. In addition, our data suggest that GRF1 and GRF3 may function as negative regulators of gene expression through their association with other transcription factors. Collectively, our data provide new insights into how GRF1 and GRF3 might coordinate the interactions between defense signaling and plant growth and developmental pathways.

## Introduction

Plants have evolved complex regulatory mechanisms to defend themselves against a wide range of biotic and abiotic stress factors. In response to pathogen infection plant cells promptly activate defense signaling, which requires considerable metabolic activity, to cope with the infection at the expense of growth-related cellular functions. Accordingly, mutant plants with constitutively activated defense responses frequently exhibit stunted growth and delayed development [1]. The growth-defense trade-off is a well-known phenomenon but the underling molecular mechanisms are elusive. In other words, the cellular factors mediating the overlaps between defense signaling and developmental pathways are unknown. In this context, growth-regulating transcription factors (GRFs) represent exciting targets to investigate the molecular mechanisms that coordinate developmental cell biology changes and defense dynamics. GRFs genes were identified in the genomes of all seed plants examined so far [2]–[5]. The GRF genes constitute a small gene family containing 9 members in *Arabidopsis thaliana* [3], 12 members in rice (*Oryza sativa*) [4] and 14 members in maize (*Zea mays*) [5]. The GRF gene family is defined by the presence of QLQ and WRC domains in the N-terminal region [3]. The QLQ domain of GRFs is involved in protein–protein interactions. The WRC domain of the GRFs contains a nuclear localization signal and a DNA-binding motif, which mediates their binding to specific *cis*-acting elements in the promoters of the target genes thereby regulating their expression [6]. It has been shown that Arabidopsis GRF1 and GRF2 act as transcriptional activators and the transactivation activity is mediated by the C-terminal region, which does not contain QLQ or WRC motifs, and through the association with the co-activator GRF-Interacting Factor (GIF) [6]. More recently, Arabidopsis GRF7 was reported to function as transcriptional repressor of osmotic stress–responsive genes by binding to the *cis*-element TGTCAGG [7]. However, the transcriptional repression activity of GRF7 requires the QLQ or WRC motifs. Taken together, these data suggest that GRF proteins can function as transcriptional activators and/or transcriptional repressor, and QLQ-binding cofactors are most likely the major determinants of the transactivation or repression activity.

Several *GRF* genes contain binding sites for microRNA396 (miR396) and thus are post-transcriptionally regulated by the activity of miR396. The induction of miR396 is frequently associated with significant decrease in *GRF* expression levels. Reduction of the expression of *GRF* genes by overexpressing miR396 suggested a role of GRFs in the development of leaves, and roots [8]–[11]. For example, miR396 accumulates preferentially in the distal part of young developing leaves and diminishes cell proliferation by inhibiting the activity of GRF2 thereby defining the ultimate number of cells in leaves [9]. Consistent with this finding, a role of GRFs in the establishment of leaf polarity was demonstrated [10]. In addition, the implication of GRFs in coordinating plant response to biotic stress has been recently suggested.

The expression of miR396-regulated *GRF* genes has been shown to be altered in response to various abiotic stress treatments including drought, salinity, low temperature, and UV-B radiation [12], [13]. Consistent with a functional role of miR396/GRFs in abiotic stress responses, GRF7 was recently demonstrated to function as a repressor of a wide range of osmotic stress-responsive genes, presumably to prevent growth inhibition under normal conditions [7]. The implication of the miR396/GRFs regulatory system in biotic stress response has been recently reported. For example, miR396 and/or *GRFs* were shown to accumulate in plants treated with the *Pseudomonas syringae* DC3000 *hrcC2* [14] and flg22 [15]. In addition, we recently discovered key functional roles of miR396-targeted *GRF1* and *GRF3* in reprogramming of root cells during cyst nematode parasitism [11], [16]. We demonstrated that *GRF1* and *GRF3* are post-transcriptionally regulated by miR396 during cyst nematode infection and that gene expression change of miR396 or its targets *GRF1* and *GRF3* significantly reduced plant susceptibility to nematode infection [16]. More importantly, we found that miR396/GRF1-GRF3 controls about 50% of the gene expression changes described in the syncytium induced by the beet cyst nematode *Heterodera schachtii* in Arabidopsis roots [16]. Collectively, these data point to roles of GRFs in controlling the overlaps between defense signaling and developmental pathways. In this study, we identified a large number of putative targets of GRF1 and GRF3 by comparing gene expression change in transgenic plants overexpressing miRNA396-resitanat version of *GRF1* (*rGRF1*) or *rGRF3* with those of the *grf1/grf2/grf3* triple mutant. Functional classification of the putative targets revealed that GRF1/3 are involved in a wide range of developmental processes and defense responses. Also, we demonstrate that GRF1/3 control the expression of other miRNA targets and may contribute to the negative regulation of their targets through association with other transcription factors. Together, our data shed lights into possible molecular mechanisms by which GRF1 and GRF3 control various developmental events and coordinate their interactions with defense responses.

## Materials and Methods

### Identification of putative targets of GRF1 and GRF3

To identify putative target genes of GRF1 and GRF3 we analyzed our recently published microarray data set (accession number GSE31593 in Gene Expression Omnibus at the National Center for Biotechnology Information, http://www.ncbi.nlm.nih.gov/geo/) [16]. In brief, we used Arabidopsis Affymetrix ATH1 GeneChips to compare the mRNA profiles of the *grf1/grf2/grf3* triple mutant and transgenic plants overexpressing miRNA396-resitanat version of *GRF1* (*rGRF1*) or *rGRF3* with those of the corresponding wild-type (Colombia-0 [Col-0] or Wassilewskija [WS]). The experiment was conducted in a completely randomized design with three independent biological replications for each of the plant types, Col-0, WS, *grf1/grf2/grf3*, *rGRF1*, and *rGRF3*. A linear model analysis of the normalized expression values was conducted for each gene across the five genetic materials and the differential expression between Col-0 and *rGRF1* or *rGRF3* and between WS and the triple mutant was determined using a false discovery rate of less than 5% and *P* value <0.05 as described in [16]. Genes showing significant reciprocal expression patterns between overexpression lines and *grf1/grf2/grf3* mutant were chosen as putative targets.

### Biological pathway identification

Biological pathway search for the putative targets of GRF1 and GRF3 was performed using NCBI/BioSystems database (http://www.ncbi.nlm.nih.gov/biosystems), which contains records from several databases including KEGG, WikiPathways, BioCyc, Reactome, the National Cancer Institute's Pathway Interaction Database and Gene Ontology (GO). We conducted the analysis to include only Arabidopsis-specific pathways. The statistical significance of gene set enrichment in each pathway was determined using Chi-square test (*P*<0.05).

### Cluster analysis and identification of tissue-specific genes

To identify tissue-specific expression of the putative targets of GRF1 and GRF3, we analyzed microarray data from the AtGenExpress expression atlas (http://www.weigelworld.org/resources/microarray/AtGenExpress) [17] and the Arabidopsis eFP Browser (http://bbc.botany.utoronto.ca/efp/cgi-bin/efpWeb.cgi) [18]. The AtGenExpress expression atlas contains gene expression data for 79 samples covering several tissues and developmental stages, while the Arabidopsis eFP Browser contains gene expression data for more than 1000 microarray data sets. The signal intensity of each probe was retrieved and logarithmically transformed (base 10) and then used to generate the heat map using MeV (Multiple Experiment Viewer) software, version 4.9 (http://www.tm4.org/mev.html).

### *Cis*-element identification in the promoter region of GRF1/3 regulated genes

The promoter region, 1,500 bp upstream of the translation initiation codon, of all GRF1/3 putative targets were retrieved from TAIR (http://www.arabidopsis.org/tools/bulk/sequences/index.jsp) and used to search for known transcription factor *cis*-regulatory elements using PLANTPAN software [19]. The frequency of each *cis*-regulatory element was determined in the positively and negatively regulated subsets of GRF1 and GRF3 putative targets. Statistical significance of the differences in the frequency of *cis* elements between the positively and negatively regulated targets was determined using χ^2^ test.

### RNA isolation and qRT-PCR analysis

For quantification of the expression levels of *GRF1* and *GRF3* in the cytokinin mutants, Wild-type Arabidopsis (ecotypes Col-0), the *ahk2 ahk3* double mutant [20] *ahp1,2,3* triple mutant [21], *type-A arr3,4,5,6* quadruple mutant [22], and *type-B arr1,12* double mutant [23] were grown on MS medium at 26°C under 16-h-light/8-h-dark conditions. Two-week-old plants were collected for RNA isolation using the method described in [24]. DNase treatment of total RNA was performed using DNase I (Invitrogen). Twenty nanograms of DNase-treated RNA were used for cDNA synthesis and PCR amplification using the Verso SYBR Green One-Step qRT-PCR Kit (Thermo Scientific) according to the manufacturer's protocol. The PCR reactions were run in an ABI 7900HT Fast Real-Time PCR System (Applied Biosystems) using the following program: 50°C for 15 min, 95°C for 15 min, and 40 cycles of 95°C for 15 s, 60°C for 30 s and 72°C for 20 s. After PCR amplification, the reactions were subjected to a temperature ramp to generate the dissociation curve to detect the nonspecific amplification products. The dissociation program was 95°C for 15 s, 50°C for 15 s, followed by a slow ramp from 50°C to 95°C. The constitutively expressed gene *Actin8* (AT1G49240) was used as an internal control to normalize gene expression levels. Quantification of the relative changes in gene expression was performed using the 2^−ΔΔCT^ method [25].

For quantification of the expression level of miR169, miR172, miR393, miR395, miR844, miR846, and miR857 in the P35S:rGRF1 and P35S:rGRF3 transgenic plants [16], total RNA was extracted from two-week-old plants with TRIzol reagent (Invitrogen) according to the manufacturer's instructions. Total RNA (5 µg) was polyadenylated and reverse transcribed using the Mir-X miRNA First-Strand Synthesis Kit (Clontech) according the manufacturer's protocol. The synthesized cDNAs then were diluted to a concentration equivalent to 40 ng total RNA µL−1 and used as a template in qPCR reactions to quantify mature miRNA expression. PCR was performed using a universal reverse primer (mRQ; supplied with the Mir-X miRNA First-Strand Synthesis Kit), complementary to the poly(T) and the mature miRNA sequences as forward primers. The miRNA-specific forward primers were extended by two A residues on the 3′ end to ensure the binding to the poly(T) region of the mature miRNA cDNA and to evade its hybridization on the miRNA precursor cDNA, as recently described [16]. The PCR reactions were run using the following program: 95°C for 3 min, and 40 cycles of 95°C for 30 s, and 60°C for 30 s. The U6 small nuclear RNA was used as an internal control to normalize the expression levels of mature miRNAs. Quantification of the relative changes in gene expression was performed as described above. Gene-specific primers used in the qPCR analysis are provided in Table S1.

### Root Length Measurements

Seeds of the transgenic lines overexpressing *rGRF1* (line 6–8) or *rGRF3* (line 11–15) described in [16], as well as wild-type Col-0 were planted vertically on modified Knop's medium supplemented or not with 100 nM N^6^-benzyladenine (BA, a cytokinin), on 4-well culture plates (BD Biosciences). The root length of at least 30 plants per line was measured as the distance between the crown and the tip of the main root in three independent experiments. Statistically significant differences between the transgenic lines and Col-0 lines were determined by unadjusted paired *t* tests (*P*<0.01).

## Results

### Identification of potential targets of GRF1 and GRF3 using microarray analysis

**Figure 1:**
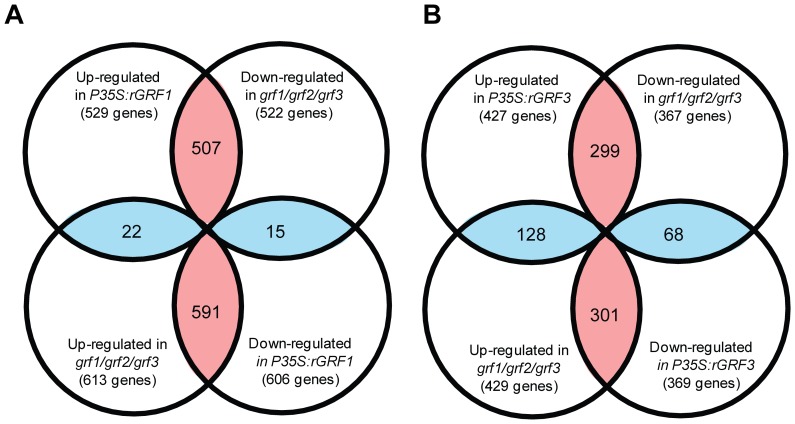
Identification of potential target genes of GRF1 and GRF3. Venn diagram comparing the overlapping differentially expressed genes between *rGRF1* and *grf1/grf2/grf3* (A) or *rGRF3* and *grf1/grf2/grf3* (B). A. Identification of potential target genes of GRF1. Out of the 1,135 overlapping genes between *grf1/grf2/grf3* triple mutant and *rGRF1*, 1,098 genes were identified as having opposite expression patterns in both lines from which 507 genes were found to be upregulated in *rGRF1* and downregulated in *grf1/grf2/grf3* triple mutant, and 591 genes were upregulated in the *grf1/grf2/grf3* mutant and downregulated in *rGRF1*. B. Identification of potential target genes of GRF3. Out of the 796 overlapping genes between *grf1/grf2/grf3* triple mutant and *rGRF3*, 600 genes were identified as having opposite expression patterns in both lines from which 299 genes were found to be upregulated in *rGRF3* and downregulated in *grf1/grf2/grf3* triple mutant, and 301 genes were upregulated in the *grf1/grf2/grf3* mutant and downregulated in *rGRF3*. Numbers in the areas highlighted in red indicate differentially expressed genes that exhibit opposite expression whereas overlapping areas highlighted in blue indicate the number of the differentially expressed genes that exhibited similar expression.

Because both GRF1 and GRF3 function as transcription factors, identifying their direct or indirect target genes will elucidate the pathways in which these transcription factors function. Recently, we used *Arabidopsis* Affymetrix ATH1 GeneChips to compare the mRNA profiles of root tissues of the *grf1/grf2/grf3* triple mutant and transgenic plants overexpressing miRNA396-resistanat version of *GRF1* (*rGRF1*) or *rGRF3* with those of the corresponding wild-type (Col-0 or Ws). We identified 3,944, 2,293 and 2,410 genes as differentially expressed in the *grf1/grf2/grf3* triple mutant, *rGRF1* and *rGRF3* plants, respectively, at a false discovery rate (FDR) of <5% and a *P* value of <0.05 [16]. In order to mine these expression data for the most likely GRF-dependent target gene candidates, we hypothesized that *bona fide* target genes of GRF1 and GRF3 likely would exhibit opposite expression patterns in the *grf1/grf2/grf3* triple mutant and *rGRF1* or *rGRF3* overexpression plants. To this end, we compared the expression patterns of the 1,135 overlapping genes between the *grf1/grf2/grf3* triple mutant and *rGRF1* and identified 1,098 genes as having opposite expression patterns in both lines (Figure 1A and Table S2). Of these 1,098 genes, 507 genes were found to be upregulated in *rGRF1* and downregulated in the *grf1/grf2/grf3* triple mutant, and 591 genes were upregulated in the *grf1/grf2/grf3* mutant and downregulated in *rGRF1* (Figure 1A and Table S2). Similarly, we compared the expression patterns of the 796 overlapping genes between *grf1/grf2/grf3* triple mutant and *rGR31*. We identified 600 genes as having opposite expression patterns in both lines, and of these, 299 genes were found to be upregulated in *rGRF3* and downregulated in the *grf1/grf2/grf3* triple mutant; 301 genes were upregulated in the *grf1/grf2/grf3* triple mutant and downregulated in *rGRF3* (Figure 1B and Table S3). We considered these 1,098 and 600 genes as putative candidate targets of GRF1 and GRF3, respectively. When we compared these two groups of genes, we identified a set of 264 genes as common putative targets of GRF1 and GRF3, leaving a unique set of 1434 genes as putative targets of GRF1 or GRF3 (Table S4). Of these 1434 potential targets, 682 are positively regulated and 752 are negatively regulated by GRF1 or GRF3, suggesting that GRF1/3 positively and negatively regulate target genes to similar extent.

### Mapping the putative targets of GRF1 and GRF3 to biological pathways reveals their function diversity

**Figure 2:**
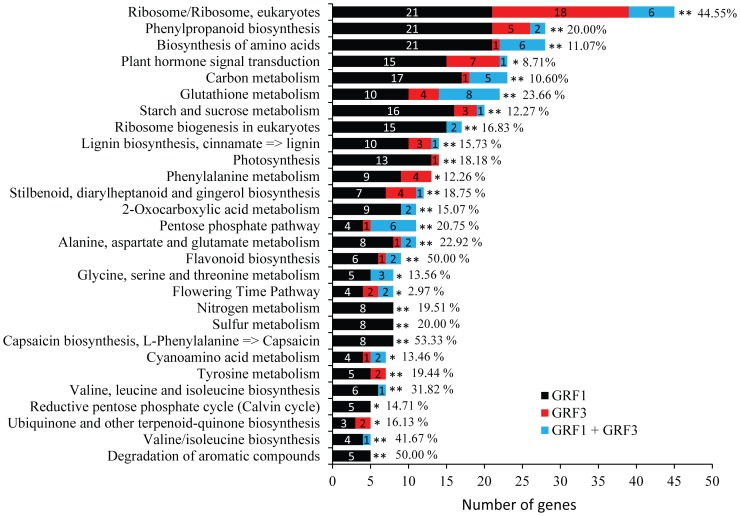
Mapping putative target genes of GRF1 and GRF3 to biological pathways. The 1434 putative target genes of GRF1/3 were subjected to NCBI/Biosystem database to identify specific biological pathways. Out of the 1434 genes, 383 were mapped to 161 organism specific pathways. We included only pathways that are represented by at least 5 genes and significantly enriched in the putative targets gene list compared with the genome. The complete description of the 161 pathways is provided in Table S5.

In order to identify specific biological pathways in which the putative targets of GRF1 or GRF3 are involved we subjected the 1434 genes to a comprehensive analysis using NCBI/Biosystem database [26]. We successfully mapped 383 genes for 161 organism specific pathways (Table S5). In Figure 2, we included only pathways that are represented by at least 5 genes and significantly enriched in the putative targets gene list compared with the genome. Genes related to flavonoid biosynthesis, degradation of aromatic compounds and capsaicin biosynthesis constitute half of the genes involved in these pathways. Also, genes involved in the biosynthesis of other secondary metabolites such as phenylpropanoid, stilbenoids, terpenoid and cyanoamino acid were also enriched in the putative targets gene list. Putative targets involved in the biosynthesis of lignin and various amino acids constitute a significant portion of these pathways. Furthermore, putative targets of GRF1 or GRF3 involved in the metabolism of glutathione, nitrogen, or sulfur are enriched in these pathways. This analysis clearly indicates the implication of these targets in a wide range of biological processes, specifically the biosynthesis of amino acid and secondary metabolites.

### GRF1 and GRF3 may regulate common targets in a tissue-specific fashion

**Figure 3:**
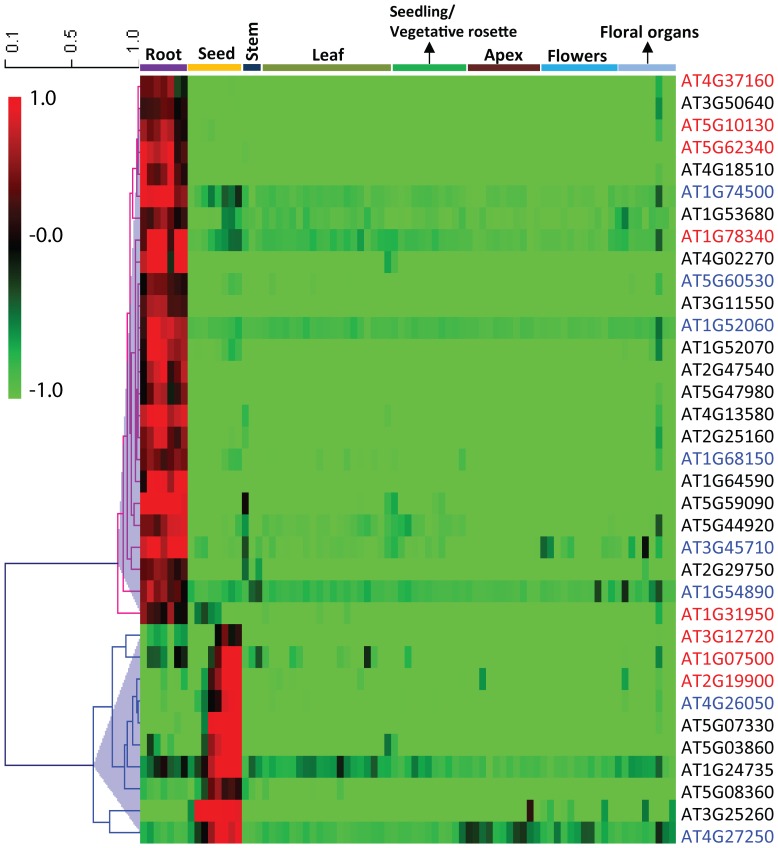
Hierarchical cluster analysis representation of root and seed-specific genes that are putative targets of GRF1 and/or GRF3. The absolute values of gene expression were logarithmically scaled (base 10) and used to generate the heat map using MeV (Multiple Experiment Viewer) software, version 4.9. Genes are represented in lines and different tissues/organs are represented in column. Red and green correspond to transcriptional upregulation and downregulation relative to the average expression level over all tissues included, respectively. Gene IDs highlighted in black, red or blue color indicate putative targets of GRF1, GRF3 or both, respectively.

To test whether the putative targets of GRF1 or GRF3 are associated with tissue specific expression patterns, the expression profiles of the 1434 putative targets were scanned across the AtGenExpress expression atlas [17], which contains 79 samples covering several tissues and developmental stages, from embryogenesis to senescence. Out of 1434 genes, we identified 130 and 13 specifically expressed in root and seed tissues, respectively. After this initial screen, the specific expression patterns of these genes were further verified by exploring a larger microarray database, the Arabidopsis eFP Browser [18], which contains more than 1000 microarray data sets. The second analysis yielded 25 and 10 genes as root and seed-specific genes, respectively (Figure 3 and Table S6). Of the 25 root-specific genes, 6 are common putative targets of both GRF1 and GRF3. Similarly, 2 genes were identified as common targets of both GRF1 and GRF3 out of the 10 seed-specific genes (Figure 3). These data suggest that GRF1 and GRF3 may regulate common targets in a tissue-specific fashion.

### GRF1 and GRF3 regulate the expression of other miRNA targets

To test whether GRF1 or GRF3 regulate other miRNA target genes, we scanned the entire set of the differentially expressed genes in *rGRF1* (2,293 genes) or *rGRF3* (2,410 genes) against all known Arabidopsis miRNAs target genes (205 genes). Interestingly, among the 2,293 genes regulated in *rGRF1*, we identified 19 genes that are post-transcriptionally regulated by 12 different miRNA gene families (Table S7). Also, among the 2,410 genes regulated in *rGRF3*, we identified 19 genes that are targets of 13 different miRNA gene families (Table S7). However, when these comparisons were narrowed to include only the putative direct targets of *GRF1* (1,098 genes) or *GRF3* (600 genes), we identified 15 genes that are targets of 7 miRNA gene families including miR169, miR172, miR393, miR395, miR844, miR846, and miR857 (Table 1). Interestingly, all targets of miR169 (7 genes) are negatively regulated by GRF1 and/or GRF3. This cross regulation seems to be organized in a coordinated manner since three out of the seven targets are co-regulated by both GRF1 and GRF3. Also, we found that GRF1 and GRF3 regulate the expression of miRNA targets in both directions. For example, targets of miR172, miR393, miR846 are positively regulated by GRF1 and/or GRF3. In contrast, targets of miR169, miR395 and miR857 are negatively regulated by GRF1 and/or GRF3.

**Table 1:** Putative targets of GRF1 or GRF3 that are post-transcriptionally regulated by miRNAs.

Gene ID	Annotation	GRF	miRNA
AT1G54160	CCAAT-binding transcription factor	GRF1	miR169
AT3G20910	CCAAT-binding transcription factor	GRF1	miR169
AT5G12840	HAP2A transcription factor	GRF1	miR169
AT1G17590	CCAAT-binding transcription factor	GRF3	miR169
AT1G72830	HAP2C transcription factor	GRF1 + GRF3	miR169
AT3G05690	HAP2B transcription factor	GRF1 + GRF3	miR169
AT5G06510	CCAAT-binding transcription factor	GRF1 + GRF3	miR169
AT3G54990	AP2 domain transcription factor	GRF3	miR172
AT4G03190	Auxin signaling F box protein 1	GRF3	miR393
AT5G10180	Sulfate transporter 68	GRF1	miR395
AT5G51270	Protein kinase family protein	GRF1	miR844
AT1G52070	Jacalin lectin family protein	GRF1	miR846
AT1G52060	Jacalin lectin family protein	GRF3	miR846
AT2G25980	Jacalin lectin family protein	GRF1 + GRF3	miR846
AT3G09220	Laccase 7	GRF3	miR857

**Figure 4:**
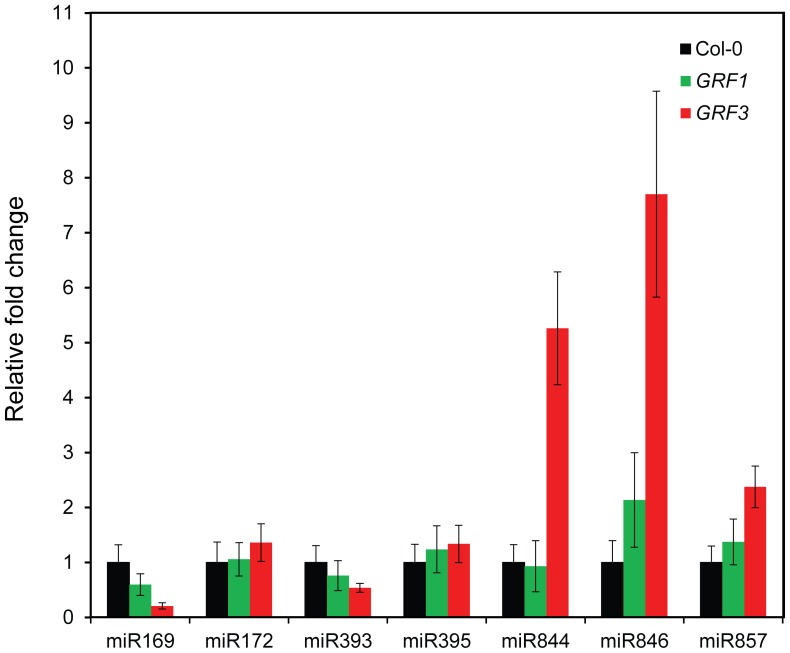
Overexpression of *rGRF1* or *rGRF3* alters the expression of other miRNAs. The expression levels of mature miR169, miR172, miR393, miR395, miR844, miR846, and miR857 were quantified in transgenic plants constitutively expressing the miR396-resistant forms of *GRF1* and *GRF3* (P35S:rGRF1 and P35S:rGRF3) using qPCR. The expression levels of mature miRNAs were normalized using *U6* snRNA as an internal control. The relative fold-change values represent changes of mature miRNA expression levels in the transgenic plants relative to the wild-type control. Data are averages of three biological samples ± SE.

Recent studies have shown that miRNA expression can be positively or negatively regulated by their targets through negative or positive feedback regulation loops [11], [27]–[31]. Therefore, we tested whether overexpression of *rGRF1* or *rGRF3* affected the expression of 7 miRNA genes (miR169, miR172, miR393, miR395, miR844, miR846, and miR857) whose targets were found to be regulated by GRF1 and/or GRF3. We used qPCR to quantify the abundance of mature miRNAs in the transgenic plants overexpressing *rGRF1* or *rGRF3* relative to wild-type Col-0. The expression levels of miR169 and miR393 were found to be downregulated both in *rGRF1* and *rGRF3* overexpression plants (Figure 4). In contrast, miR844, miR846 and miR857 showed predominant upregulation in the transgenic plants overexpression *rGRF3*, and to lesser extent in the transgenic plants overexpression *rGRF1* (Figure 4). miR172 and miR395 showed little or no changes in the transgenic plants (Figure 4). These data clearly demonstrate that GRF1 and GRF3 can contribute to the negative or positive regulation of other miRNA genes through altering the expression of their targets.

Because we previously found that *GRF1* and *GRF3* change their expression in the syncytium induced by *H. schachtii* [16], it was of interest to test whether the 15 miRNA targets regulated by GRF1 and/or GRF3 are differentially expressed in the syncytium. Interestingly, these entire target genes were found to be differentially expressed in the syncytium induced by *H. schachtii* according to microarray analysis reported by [32]. However, when these 15 target genes were compared with those reported to be differentially expressed in the giant cells induced by the root-knot nematode *Meloidogyne incognita* [33], none of these genes were found to be overlapped. These data suggest that the regulation of miRNA targets by GRF1/3 is specific to the syncytial cells.

### GRF1 and GRF3 regulate cytokinin-responsive genes

**Figure 5:**
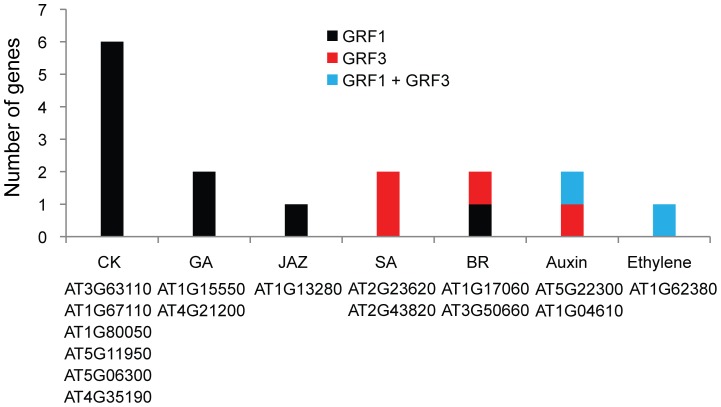
Putative targets of GRF1/3 are involved in hormone biosynthesis pathways. Sixteen potential targets of GRF1/3 are implicated in the biosynthesis of various hormone pathways with cytokinin biosynthesis genes being the most abundant.

Our examination of the GRF-regulated targets for genes involved in hormone biosynthesis pathways led to the identification of a set of genes that are involved in the biosynthesis of cytokinin (6 genes), brassinosteroid (2 genes), auxin (2 genes), gibberellin (2 genes) salicylic acid (2 genes), ethylene (1 gene), and jasmonic acid (1 gene) (Figure 5). The abundance of cytokinin biosynthesis genes in this gene set prompted us to speculate that cytokinin-responsive genes could be also regulated by GRF1/3. To test this hypothesis, the 2,293 genes regulated by GRF1 were compared with the golden list of the cytokinin-responsive genes [34]. Out of the 226 cytokinin-responsive genes, 61 were identified as overlapping with GRF1-regulated genes. Similarly, 43 of the cytokinin-responsive genes overlapped with GRF3-regulated genes. After eliminating duplicates, a total of 92 (41%) cytokinin-responsive genes were identified as overlapping with the GRF1/3-regulated genes (Table S8). When these analyses were conducted to include only the potential targets of GRF1/3 (1434 genes), we identified 48 (21%) of the cytokinin-responsive genes as overlapping (Table 2). These data suggest that GRF1 and GRF3 play major role in controlling gene expression changes of cytokinin-responsive genes.

**Table 2:** Cytokinin-responsive genes that are identified as putative targets of GRF1 or GRF3.

Gene ID	Annotation
AT2G01890	PAP8 (PURPLE ACID PHOSPHATASE PRECURSOR)
AT1G13420	sulfotransferase family protein
AT5G63450	CYP94B1 (cytochrome P450, family 94, subfamily B, polypeptide 1)
AT5G10580	Unknown protein
AT5G03380	Heavy-metal-associated domain-containing protein
AT2G17820	HISTIDINE KINASE 1
AT1G59940	ARR3 (RESPONSE REGULATOR 3)
AT5G38020	S-adenosyl-L-methionine:carboxyl methyltransferase family protein
AT1G67110	CYP735A2 (cytochrome P450, family 735, subfamily A, polypeptide 2)
AT1G15550	GA4 (GA REQUIRING 4); gibberellin 3-beta-dioxygenase
AT1G47400	Unknown protein
AT1G14960	Major latex protein-related/MLP-related
AT5G04120	Phosphoglycerate/bisphosphoglycerate mutase family protein
AT3G10960	Xanthine/uracil permease family protein
AT2G17500	Auxin efflux carrier family protein
AT4G21120	AAT1 (CATIONIC AMINO ACID TRANSPORTER 1)
AT1G69040	ACR4 (ACT REPEAT 4); amino acid binding
AT3G57040	ARR9 (RESPONSE REACTOR 4); transcription regulator
AT5G47980	Transferase family protein
AT1G67030	ZFP6 (ZINC FINGER PROTEIN 6)
AT5G05790	Myb family transcription factor
AT4G19030	NLM1 (NOD26-like intrinsic protein 1;1)
AT2G34610	Unknown protein
AT3G15990	SULTR3;4; sulfate transmembrane transporter
AT3G59670	Unknown protein
AT2G23170	GH3.3; indole-3-acetic acid amido synthetase
AT1G64590	Short-chain dehydrogenase/reductase (SDR) family protein
AT3G21670	Nitrate transporter (NTP3)
AT5G60890	ATMYB34
AT2G38750	ANNAT4 (ANNEXIN ARABIDOPSIS 4)
AT4G34950	Nodulin family protein
AT2G46660	CYP78A6 (cytochrome P450, family 78, subfamily A, polypeptide 6)
AT5G01740	Similar to SAG20 (WOUND-INDUCED PROTEIN 12)
AT2G25160	CYP82F1 (cytochrome P450, family 82, subfamily F, polypeptide 1)
AT2G36950	Heavy-metal-associated domain-containing protein
AT4G23750	CRF2 (CYTOKININ RESPONSE FACTOR 2)
AT5G64620	Invertase inhibitors AtC/VIF2
AT3G29250	Oxidoreductase
AT1G49470	Unknown protein
AT5G65210	TGA1
AT5G47990	CYP705A5 (cytochrome P450, family 705, subfamily A, polypeptide 5)
AT4G29700	Type I phosphodiesterase/nucleotide pyrophosphatase family protein
AT1G78000	SULTR1;2 (SULFATE TRANSPORTER 1;2)
AT3G45710	Proton-dependent oligopeptide transport (POT) family protein
AT4G25410	basix helix-loop-helix family protein
AT5G48000	CYP708A2 (cytochrome P450, family 708, subfamily A, polypeptide 2)
AT5G26220	ChaC-like family protein
AT1G66800	Cinnamyl-alcohol dehydrogenase family/CAD family

**Figure 6:**
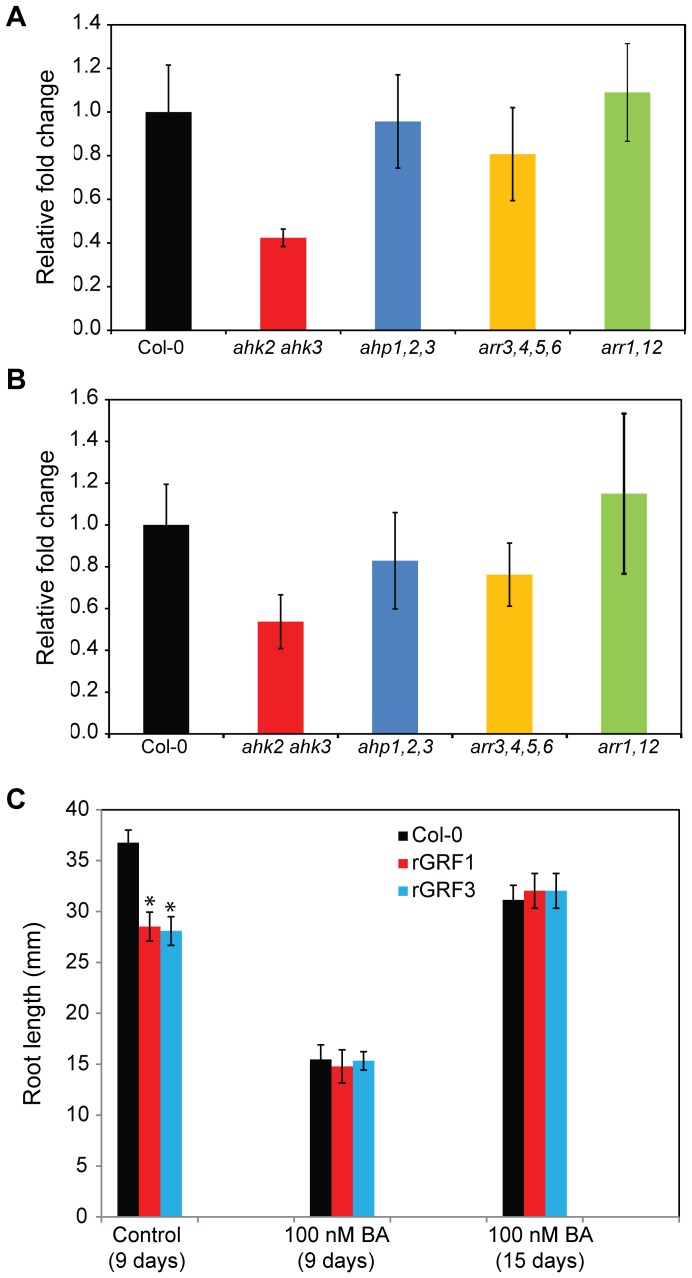
GRF1 and GRF3 regulate cytokinin signaling. A and B, GRF1 and GRF3 may contribute to the activity of cytokinin receptors. The expression levels of *GRF1* (A) and *GRF3* (B) were quantified by qPCR in various cytokinin signaling mutants including the *ahk2 ahk3* double mutant, *ahp1,2,3* triple mutant, type-A *arr3,4,5,6* quadruple mutant and type-B *arr1,12* double mutant. *GRF1* and *GRF3* showed significant downregulation in the *ahk2 ahk3* double mutant. The expression levels of *GRF1* and *GRF3* were normalized using *actin8* as an internal control. The relative fold-change values represent changes of *GRF* expression levels in the mutant lines relative to the wild-type (Col-0). Data are averages of three biological samples ± SE. C, Exogenous application of cytokinin rescued the short-root phenotype of *rGRF1* and *rGRF3* overexpression lines. Homozygous T3 lines overexpressing *rGRF1* (line 6–8), or *rGRF3* (line 11–15) as well as the wild-type Col-0 were grown vertically on modified Knop's medium supplemented or not with 100 nM BA and root lengths were measured 9 and 15 days after planting. Root length values are averages of at least 30 plants ± SE. Mean values significantly different from that of the wild type as determined by unadjusted paired t tests (P<0.01) are denoted by an asterisk.

In plants, cytokinin is perceived through a multi-step phosphorelay pathway. Based on the current model in Arabidopsis, three histidine Kinases, AHK2, AHK3 and AHK4 have been identified as transmembrane cytokinin receptors. These receptors transfer the signal via Arabidopsis histidine phosphotransfer proteins (AHPs) to the nucleus, activating two types of primary Arabidopsis response regulators (ARRs), known as type-A and type-B response regulators [35]. To provide direct evidence for the connection between GRF1/3 and cytokinin signaling, we measured the expression levels of *GRF1* and *GRF3*, using qPCR, in several cytokinin signaling mutants including the *ahk2 ahk3* double mutant, *ahp1,2,3* triple mutant, type-A *arr3,4,5,6* quadruple mutant and type-B *arr1,12* double mutant. Data from three biological replicates revealed that the expression levels of *GRF1* and *GRF3* are significantly changed in the *ahk2 ahk3* double mutant, showing at least twofold down-regulation in the mutant relative to wild-type plants (Figure 6A and B). In contrast, the expression levels of *GRF1* and *GRF3* were not significantly altered in the *ahp1,2,3*, type-A *arr3,4,5,6* or type-B *arr1,12* mutant lines (Figure 6A and B). These data support a role for GRF1 and GRF3 in the regulation of cytokinin receptors.

One of the main morphological defects in the transgenic plants overexpressing *rGRF1* or *rGRF3* is the short-root phenotype [16]. Because cytokinin regulates the root meristem activity, root size and overall root length [36], therefore, it was of interest to examine whether the short-root phenotype in the *rGRF1* and *rGRF3* is mediated by cytokinin. To this end, homozygous T3 plants overexpressing *rGRF1* (line 6–8), or *rGRF3* (line 11–15) as well as the wild-type (Col-0) were grown vertically on modified Knop's medium supplemented or not with cytokinin in the form of benzyladenine (BA) at the concentration of 100 nM. Without exogenous application of cytokinin, the transgenic plants overexpressing *rGRF1* or *rGRF3* developed statistically significant shorter roots than the wild-type Col-0 at 9 days after planting (Figure 6C), confirming our previously published data [16]. Because exogenous application of cytokinin reduces root size and growth, we decided to compare the root length of the transgenic plants overexpressing *rGRF1* or *rGRF3* with Col-0 at 9 and 15 days after planting on modified Knop's medium supplemented with 100 nM BA. Interestingly, at both time points, the root lengths of the transgenic plants were found to be very similar to that of the Col-0 and no statistically significant differences were detected (Figure 6C). These results provide further support that GRF1 and GRF3 play key role in regulating gene expression changes of cytokinin-responsive genes.

### Several transcription factor gene families are putative targets of GRF1/3

**Figure 7:**
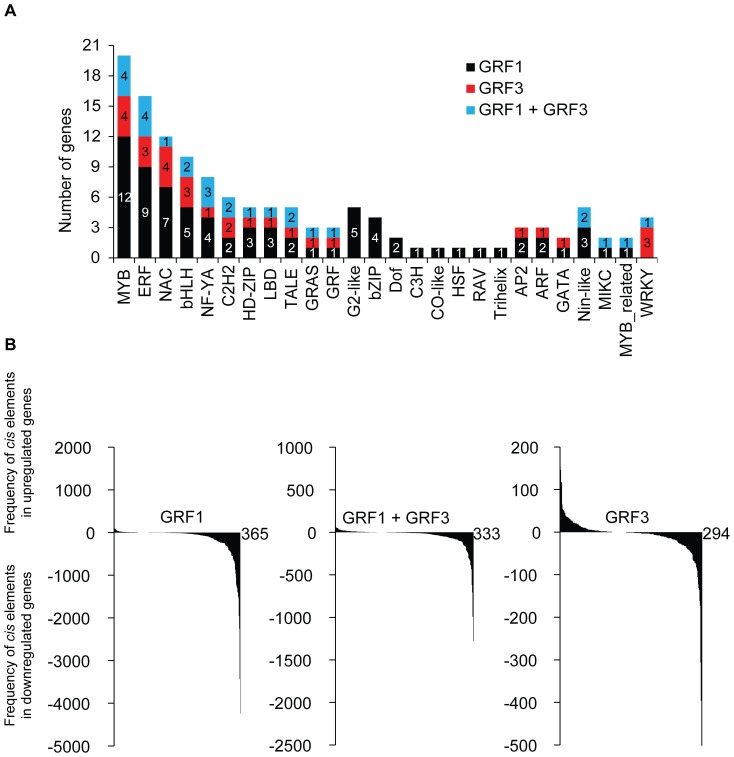
GRF1 and GRF3 may function as negative regulators of gene expression through their association with other transcription factors. A. Histogram showing the number of genes in different transcription factor families that are identified as putative targets of GRF1 or GRF3. B. The frequency of various transcription factor *cis* elements was quantified in the promoters (1,500 bp upstream of the translation start codon) of upregulated putative targets of GRF1, GRF3 or both versus downregulated genes using PlantPan software [19]. For each *cis* element (x axis), the differences in the frequency between upregulated and downregulated targets (y axis) were calculated and used in the plot.

Careful examination of the potential targets of GRF1/3 revealed that high number of these targets code for transcription factors (Figure 7A). Transcription factors of the MYB, ERF NAC, bHLH and NF-YA gene families are highly represented. Interestingly, we identified four bZIP/TGA transcription factor genes (*TGA1, 3, 4* and *7*) that are specifically regulated by GRF1. These genes are members of clade I (*TGA1* [At5g65210] and *TGA4* [At5g10030]) and clade III (*TGA3* [At1g22070] and *TGA7* [At1g77920]). Functional characterization of clade I and III TGA factors has established an essential role in the regulation of pathogenesis-related genes and disease resistance [37]–[39]. In addition, we identified several MYB transcription factors as potential targets of GRF1 (*MYB58* [AT1G16490], *MYB63* [AT1G79180] and *MYB43* [AT5G16600]), which are involved in the regulation of secondary cell wall formation [40], [41]. Consistent with this finding, genes with cell-wall related functions constitute 10 and 15% of the differentially expressed genes identified in the transgenic plants overexpression *GRF1* or *GRF3*, respectively. Another interesting finding that may connect the function of GRF1 and GRF3 to a wide range of developmental processes and biotic stress tolerance is that several ethylene-responsive element-binding factors (*ERFs*) were identified as putative targets of GRF1 and GRF3. ERFs impact a number of developmental processes and are also function in plant adaptation to biotic and abiotic stresses [42]–[44].

### GRF1 and GRF3 may function as negative regulators of gene expression through their association with other transcription factors

Because GRF1/3 contain the QLQ protein/protein interaction domain, we hypothesized that other transcription factors may form a complex with GRF1/3 and facilitate the binding of GRF1/3 to specific binding motifs in the promoter of their putative targets. Therefore, we searched for known *cis*-elements that would be involved in the transcriptional regulation of all putative target genes of GRF1 and GRF3 in a 1.5 kb promoter region upstream of the translation start codon using PlantPan software [19]. We identified 382 and 361 *cis* elements in the promoters of the putative targets of GRF1 and GRF3, respectively (Table S9). Interestingly, when these *cis* elements were compared to identify common elements, the majority of these elements (357) were found to be common in the promoters of the putative targets of GRF1 and GRF3. These data suggest that both GRF1 and GRF3 may employ similar mechanisms in regulating the expression of their targets, consistent with the redundant function of these two transcription factors. In addition, we tested the distribution and frequency of these *cis* elements in the positively and negatively regulated targets of GRF1 (834 genes), GRF3 (336 genes) and both (264 genes). While these *cis* elements are equally distributed between up and downregulated genes, their frequency is much higher in the downregulated genes (Figure 7B), suggesting that GRF1 and GRF3 may function as negative regulators of gene expression through their association with other transcription factors.

## Discussion

Despite the efforts to assign the biological processes regulated by GRFs during plant development, very limited number of target genes have been identified and characterized to date [6], [7]. One of the most common approaches to identify target genes of the transcription factors involves comparison of the genome-wide transcript profiles of transgenic plants overexpressing transcription factors and the corresponding wild types allowing the identification of genes that are significantly altered as a result of the increased expression of the transcription factors [45], [46]. An alternative approach relies on the comparison between the transcriptome of mutants and wild-type plants [47]–[49]. In the current study, we combined both approaches to identify potential target genes of GRF1 and GRF3. We retained only genes showing opposite expression between *grf1/grf2/grf3* triple mutant and *rGRF1* or *rGRF3* in order to exclude genes whose expression is altered as artifactual effects of the ectopic overexpression and do not reflect authentic roles of the overexpressed transcription factors. Using this approach we identified 1,098 and 600 genes as putative targets of GRF1 and GRF3, respectively. These numbers are relatively low compared with the total number of genes regulated by GRF1 (1,098 genes out of 2293, 47.9%) or GRF3 (600 genes out of 2410, 24.9%), suggesting that the greater part of these genes are indirectly regulated. The indirect regulation of downstream genes could be through the transcription control mediated by transcription factors or proteins with binding activity among those directly regulated by GRF1 or GRF3. Consistent with this interpretation, genes coding for transcription factors or proteins with binding activity represent up to 39% of the GRF1- potential direct target genes and up to 35% of the GRF3- potential direct targets. The enrichment of transcription factors belonging to Myb, ERF, NAC, bHLH, NY-YA, and C2H2 transcription factor family proteins in GRF1 or GRF3- potential direct target genes suggests key roles of these transcription factors in initiating transcriptional cascades, thereby extending the effects of GRF1 or GRF3 on downstream signaling pathways.

Transcription factors can positively or negatively regulate the expression of their target genes [50]. Our data point to the possibility that GRF1/3 may function as transcriptional repressors since more than half of the GRF1/3 targets are negatively regulated. Initially, members of the GRF gene family have been shown to function as transcriptional activators and this transactivation function involves the C-terminal region [6]. More recently, GRF7 was found to function as transcriptional repressor through its N-terminal QLQ and WRC motifs [7]. Because GRF proteins contain the QLQ protein–protein interaction domain, it is possible that GRF1/3 contribute to the negative regulation of their targets through their association with other transcription factors. This hypothesis is developed based on our data showing that the frequency of known *cis* elements is more abundant in the negatively regulated targets relative to the upregulated targets (Figure 7B). However, we don't rule out the possibility that GRF1/3 may function as transcriptional repressors through their biding to specific *cis* motifs.

Functional classification of the potential targets of GRF1/3 placed these two transcription factors as molecular links connecting defense signaling to plant growth and developmental pathways. Previously, we reported a key role for GRF1/3 in plant response to nematode infection [16]. In the current analysis, the anticipated roles of GRF1/3 in defense responses is further illuminated by identifying crucial factors that are involved in defense response and disease resistance. Four bZIP/TGA transcription factors genes (*TGA1, 3, 4* and *7*) were identified as potential targets of GRF1. *TGA1* and *TGA4*, which belong to clade I are positively regulated, whereas *TGA3* and *TGA7*, which belong to clade III are negatively regulated by GRF1. Characterization of Arabidopsis T-DNA insertion mutants indicated that clade I TGA factors contribute to basal disease resistance and this contribution is most likely independent of NPR1 [39], [51], [52]. In contrast, NPR1 stimulates the DNA binding of the clade III factors (TGA3 and TGA7) to the promoter of *PR1* in a SA-dependent manner [39], [53]–[56]. It seems that GRF1 regulates the synergistic interactions between clade I and III TGA factors during plant response to pathogen infection by oppositely regulating the expression of genes belonging to both groups. Similar to clade I, clade III factor TGA3 is required for basal resistance [51] as well as for a novel form of cytokinin-induced resistance against virulent *P. syringae* [57]. Cytokinin-induced resistance may be an additional mechanism by which GRF1/3 control pathogen infection. Consistent with this speculation we found that GRF1/3 regulate 92 genes (41%) of the cytokinin-responsive genes from which 48 genes (21%) were identified as putative targets. Our data suggest that the potent control of GRF1/3 over cytokinin-responsive genes could be through targeting these genes directly as well as genes involved in cytokinin biosynthesis and signaling pathways. This suggestion was further supported by our data showing a significant down regulation of *GRF1* and *GRF3* in the cytokinin receptor *ahk2 ahk3* double mutant and that exogenous application of cytokinin rescued the short-root phenotype of the transgenic plants overexpressing *rGRF1* or *rGRF3* (Figure 6). Cytokinins are fundamental hormones for the proper growth and development of the plants [58] and also play critical roles in plant-pathogen interaction as many plant pathogens secrete cytokinins or promote cytokinin accumulation in host plants [57], [59]–[61]. We conclude that targeting cytokinin-responsive and/or biosynthesis genes by GRF1/3 seems to be one of the main mechanisms employed by these two transcription factors to synchronize developmental processes and defense responses during pathogen infection.

Another interesting finding that could explain the coordination between developmental processes and defense responses mediated by GRF1/3 is that several ethylene-responsive element-binding factors (ERFs) are identified as putative targets of GRF1/3. ERFs constitute a plant-specific transcriptional factor superfamily of 147 members in Arabidopsis [62], influence a number of developmental processes, and are also involved in plant response to biotic stress [63]–[65]. It might be relevant to mention that several ERFs we identified as putative targets of GRF1/3 are implicated in defense responses. For example ERF5 (AT5G47230) plays vital role in phytotoxin-triggered programmed cell death [65] and in regulating both stress tolerance and leaf growth inhibition [66]. In addition, ERF2 (At5g47220) induces high levels of defense gene expression and enhances plant resistance to *Fusarium oxysporum* when overexpressed in Arabidopsis [67], [68]. Furthermore, four *ERFs* (AT1G28370, AT2G33710, AT3G50260 and AT5G47220) identified as potential targets of GRF1/3 were found to be highly upregulated in response to chitin, a plant-defense elicitor [69]. These transcription factors may regulate gene expression downstream of chitin-activated defense signaling pathways in association with GRF1/3. Interestingly, WRKY33 was identified as potential direct target of GRF1 and GRF3. WRKY33 is a pathogen-inducible transcription factor, functions downstream of MPK3/MPK6 in controlling the accumulation of camalexin, the major phytoalexin in Arabidopsis. WRKY33 binds directly to the promoter of *PAD3*, which catalyzes the last conversion step of camalexin pathway [70], [71]. It is intriguing to find that out of the ten genes known to be involved in the camalexin biosynthetic process, 5 were identified as putative targets of GRF1/3 including *MKK9, MPK3, PAD3* and *NAC* domain-containing protein 42 in addition to WRKY33. These data suggest that GRF1/3 may contribute significantly to the regulation of camalexin biosynthetic genes and hence defense responses.

Plants respond to invading pathogens by activating various metabolic pathways including induction of an array of secondary metabolites with antimicrobial properties as an integral part of plant disease resistance [72], [73]. Regulating the activity of various secondary metabolite pathways appears to be another way by which GRF1/3 regulate defense responses. Our analysis revealed that several genes involved in the biosynthesis of several secondary metabolites including capsaicin, phenylpropanoid, stilbenoids, terpenoid and cyanoamino acid constitute a significant portion of the GRF1/3 putative targets. Unlike primary metabolites, secondary metabolites are not directly involved in the normal growth, development, or reproduction of the plants. However, they frequently play an important role in plant immunity by controlling the entry and/or development of the pathogens into plant cells and tissues as these metabolites can be secreted and delivered directly at the plant-pathogen interface [73], [74]. For example, stilbenoids can function as antimicrobial compounds and accumulate as phytoalexins following pathogen infection [73]. Constitutive expression of a grapevine stilbene-synthase gene in alfalfa resulted in increased plant resistance to the leaf spot pathogen *Phoma medicaginis* [75]. Phenylpropanoids serve as precursors for several compounds essential for disease resistance and their association with active defense response are well-known [76]–[78]. Terpenoids are the biggest and most diverse class of phytochemicals and recent data demonstrate that their accumulation in plant tissues can modify plant interactions with various pathogens [79].

Molecular links between defense and developmental pathways are believed to mediate and control the cross talk between various signaling pathways. This was clearly demonstrated by our data showing that GRF1/3 regulate other miRNA target genes that are involved in various cellular processes including flowering, auxin signaling, and copper and sulfate homeostasis (Table 1). Interestingly, this regulation was extended to include the expression of these miRNAs. As shown in Figure 4, the expression levels of seven miRNAs (miR169, miR172, miR393, miR395, miR844, miR846, and miR857) were altered in the transgenic plants overexpressing *GRF1* or *GRF3*. It is unlikely that GRF1 and GRF3 directly impact the expression of these miRNAs. Most likely, the expression of these miRNAs are altered as a results of positive or negative feedback regulation loops between these miRNAs and their targets that are regulated by GRF1 and/or GRF3. This assigns new and unexpected roles for these transcription factors in regulating the crosstalk between miRNA signaling networks. Our finding that GRF1 and GRF3 regulate the expression of all targets of miR169 (7 genes) from which 3 are co-regulated by both GRF1 and GRF3 suggests that the cross regulation is organized in a coordinated manner. Thus, GRF1/3 may fine tune the expression levels of co-regulated genes and members of multigene families with concomitant biological functions. Consistent with this hypothesis, several genes involved in flowering control (AT3G20910, AT5G12840, AT1G72830, AT1G17590, AT3G05690 and AT3G54990) and negatively regulated by miR169 or miR172 [31], [80], [81] were identified as putative targets of GRF1/3. Similarly, genes involved in auxin signaling such as auxin response factors, NAC domain-containing proteins, and auxin signaling F box protein1, which are negatively regulated by miR167, miR164 and miR393 [82]–[84], respectively, are also regulated by GRF1 or GRF3.

It is of interest to find that GRF1 and 3 regulate the expression of their putative targets in a tissue-specific manner. Identifying a subset of putative targets of GRF1/3 that are specifically expressed in roots is consistent with the abundant expression of *GRF1/3* in various root-tissue types and that overexpression of *GRF1* or *GRF3* impacts root growth and development [16]. Also, several recent reports support a role of GRF family members in floral organ development [85]–[88]. Our identification of several seed-specific genes as putative targets of GRF1/3 in the current study could illuminate the molecular events controlled by GRFs and required for precise floral organ initiation and development.

In conclusion, our data provide new insights into the molecular events by which GRF1/3 directly or indirectly regulate a variety of biological processes to formulate a decisive coordination between plant growth and defense responses. While direct proof is lacking, GRF1/3 may function not only as transcriptional activators or transcriptional repressors but also oppositely regulate genes that share common function or even genes that belong to the same gene family. This bifunctional activity, which reveals an unexpected degree of complexity of GRF1/3 in the regulation of their targets, may count among the main characteristics of key genes linking plant growth and developmental pathways to defense signaling.

## Supporting Information

Table S1 **Primer sequences used in this study.**
(XLSX)

Table S2 **List of 1,098 differentially expressed genes showing opposite expression in the** ***grf1/grf2/grf3*** **triple mutant and** ***rGRF1*** **lines.**
(XLSX)

Table S3 **List of 600 differentially expressed genes showing opposite expression in the** ***grf1/grf2/grf3*** **triple mutant and** ***rGRF3*** **lines.**
(XLSX)

Table S4 **List of 1,434 genes identified as unique putative target genes of GRF1 and GRF3.**
(XLSX)

Table S5 **Biological pathway description of 383 putative targets of GRF1 or GRF3.**
(XLSX)

Table S6 **Putative targets of GRF1 or GRF3 showing root and seed-specific expression.**
(XLSX)

Table S7 **List of miRNA target genes that are identified as differentially expressed in the** ***rGRF1*** **or** ***rGRF3*** **transgenic plants.**
(XLSX)

Table S8 **List of cytokinin-responsive genes that are identified as differentially expressed in the** ***rGRF1*** **or** ***rGRF3*** **transgenic plants.**
(XLSX)

Table S9 **List of the** ***cis*** **elements that are identified in the promoters of the putative targets of GRF1 and GRF3.**
(XLSX)
